# TranscriptomeBrowser 3.0: introducing a new compendium of molecular interactions and a new visualization tool for the study of gene regulatory networks

**DOI:** 10.1186/1471-2105-13-19

**Published:** 2012-01-31

**Authors:** Cyrille Lepoivre, Aurélie Bergon, Fabrice Lopez, Narayanan B Perumal, Catherine Nguyen, Jean Imbert, Denis Puthier

**Affiliations:** 1 TAGC UMR_S 928, Inserm, Parc Scientifique de Luminy, Marseille, France; 2 Université de la Méditerranée, AMU, Parc Scientifique de Luminy, Marseille, France; 3 TGML, IBiSA Platform, Parc Scientifique de Luminy, Marseille, France; 4 Translational Science, Eli Lilly and Company, Lilly Corporate Center, Indianapolis, USA; 5 ESIL, Universités de Provence et de la Méditerranée, Parc Scientifique de Luminy, Marseille, France

## Abstract

**Background:**

Deciphering gene regulatory networks by *in silico *approaches is a crucial step in the study of the molecular perturbations that occur in diseases. The development of regulatory maps is a tedious process requiring the comprehensive integration of various evidences scattered over biological databases. Thus, the research community would greatly benefit from having a unified database storing known and predicted molecular interactions. Furthermore, given the intrinsic complexity of the data, the development of new tools offering integrated and meaningful visualizations of molecular interactions is necessary to help users drawing new hypotheses without being overwhelmed by the density of the subsequent graph.

**Results:**

We extend the previously developed TranscriptomeBrowser database with a set of tables containing 1,594,978 human and mouse molecular interactions. The database includes: (i) predicted regulatory interactions (computed by scanning vertebrate alignments with a set of 1,213 position weight matrices), (ii) potential regulatory interactions inferred from systematic analysis of ChIP-seq experiments, (iii) regulatory interactions curated from the literature, (iv) predicted post-transcriptional regulation by micro-RNA, (v) protein kinase-substrate interactions and (vi) physical protein-protein interactions. In order to easily retrieve and efficiently analyze these interactions, we developed In-teractomeBrowser, a graph-based knowledge browser that comes as a plug-in for Transcriptome-Browser. The first objective of InteractomeBrowser is to provide a user-friendly tool to get new insight into any gene list by providing a context-specific display of putative regulatory and physical interactions. To achieve this, InteractomeBrowser relies on a "cell compartments-based layout" that makes use of a subset of the Gene Ontology to map gene products onto relevant cell compartments. This layout is particularly powerful for visual integration of heterogeneous biological information and is a productive avenue in generating new hypotheses. The second objective of InteractomeBrowser is to fill the gap between interaction databases and dynamic modeling. It is thus compatible with the network analysis software Cytoscape and with the Gene Interaction Network simulation software (GINsim). We provide examples underlying the benefits of this visualization tool for large gene set analysis related to thymocyte differentiation.

**Conclusions:**

The InteractomeBrowser plugin is a powerful tool to get quick access to a knowledge database that includes both predicted and validated molecular interactions. InteractomeBrowser is available through the TranscriptomeBrowser framework and can be found at: http://tagc.univ-mrs.fr/tbrowser/. Our database is updated on a regular basis.

## Background

In the last decade, the advent of high throughput technologies led to the emergence of the systems biology era and prompted the research community to systematically define the expression levels of mRNAs and micro-RNA (miRNAs) through thousands of cell and tissues under physiological and pathological conditions [1]. Now, one of the crucial issues is to define the biological mechanisms that drives genes expression with the ultimate goal of reverse-engineering gene regulatory networks (GRN) as a whole in order to predict the system outcome under molecular perturbations.

One current limit for biologists interested in mining regulatory information or for bioinformaticians interested in creating regulatory maps for modeling, is that this information is scattered over the Internet under various formats making it difficult to handle. Thus one needs to create a unified database that would list known and predicted molecular interactions. This information can be obtained from different sources: (i) from the literature, (ii) from large-scale experimental methods that allow genome-wide profiling of transcription factors (TFs) binding sites to DNA or (iii) from DNA sequence analysis, by searching 3'UTR regions for miRNA specific motifs or by scanning gene promoters with transcription factor specific position weight matrices (PWMs). In the latter case, the use of comparative genomics is known to greatly improve predictions of functional TF binding sites by limiting the number of false positives (though increasing false negative rate) [2,3]. Another limit of GRN analysis is the intrinsic complexity of the data. In this regard, several graph-based tools have been developed to draw a global picture of the putative interactions taking place in the biological context of interest (for a review, see reference [4]). In these, genes or proteins appear as nodes in a graph, and functional relations (physical/regulatory interactions) are represented as edges connecting the corresponding entities. The topology of the subsequent network can later be analyzed using advanced tools such as Cytoscape [5]. However, as data integration is a challenge that requires to map various types of evidence onto a set of stable gene ids, most applications are oriented toward a single data type (mostly regulatory or physical interactions, see table 1 for an overview) [6-10] Moreover, another challenge is the development of graph-based tools producing clear, meaningful and integrated visualizations from which users can draw new hypotheses without being overwhelmed by the density of the presented graphic information. In this regard, the Cytoscape plug-in "Cerebral" proposes an intuitive visualization method through a "cell compartment-based layout" that shows interacting proteins on a layout resembling "traditional" signalling pathway/system diagrams [11].

**Table 1:** A comparison of web tools dedicated to molecular interactions.

		MIR@NT@N	**STRING**^**d**^	**MotifMap**^**e**^	GeneMANIA	**APID**^**f**^	InnateDB	InteractomeBrowser
	Physical protein protein interactions	-	+	+	+	+	+	+
	Computationally predicted TF targets^a^	+	-	+	-	-	-	+
	Experimentally observed TF targets^b^	-	-	-	-	-	-	+
**Database content**	Predicted miRNA targets	+	-	-	-	-	-	+
	Regulatory interactions from literature	-	+	-	-	-	-	+
	Biological pathways	-	+	-	+	-	-	-
	Inferred functional interactions^c^	-	+	-	+	-	-	-

	Batch query	+	+	-	+	-	-	+
**Build-in graph visualizer**	add/remove/hide inter-actors and interactions	-	-	-	-	+	-	+
	Movable nodes	-	+	ND	+	+	+	+
	Compartment-based layout	-	-	-	-	-	+	+

The table provides an overview of the types of molecular interactions and of the functionalities offered by representative web tools previously published. Informations were obtained from latest articles describing the servers. The presence or absence of the corresponding features is denoted by + or - respectively.

^a ^Refers to bioinformatic prediction of TFBSs using PWMs.

^b ^Refers to results from large-scale experimental methods that profile the binding of TFs to DNA at the genome-wide level (*e.g*.; ChIP-Seq, ChIP-chip, ...).

^c ^Refers to computational methods that aggregate various informations (*e.g*.; expression, genomic distance, conservation) to infer functional interactions.

^d ^Search Tool for the Retrieval of Interacting Genes/Proteins

^e ^MotifMap visualizer was not available during our tests. Informations related to the visualizer were obtained from documentation.

^f ^Agile Protein Interaction DataAnalyzer

Here, we sought to create a compendium of predicted and validated molecular interactions in human and mouse. First, we used a large collection of PWMs obtained from TRANSFAC (n = 523), JASPAR (n = 303) and UNIPROBE (n = 387) to search, in gene promoter regions, for candidate transcription factor binding sites (TFBSs) conserved over human, mouse, rat and dog genomes [12-14]. Overall, our analysis of these PWMs corresponding to 347 human and 475 mouse transcription factors (TFs) provides a systematic overview of gene regulation in the human and mouse. Data generated in this study were next integrated with a large set of molecular interactions from various sources including (i) potential protein/DNA interactions derived from ChIP-seq experiments (ChIP-X database), (ii) curated regulatory interactions obtained from the literature (OregAnno, LymphTF-DB), (iii) predicted miRNA/targets interactions (TargetScan) (iv) protein kinase-substrate interactions derived from multiple online sources (KEA) and (v) physical protein-protein interactions obtained from HPRD, Reactome and various databases of the IMEx consortium [15-30]. Informations related to these interactions were stored as MySQL tables that were integrated in the back-end database of TranscriptomeBrowser, our previously published microarray datamining software [31]. Finally, we developed InteractomeBrowser (IBrowser) as a plugin for TranscriptomeBrowser. IBrowser was developed using the prefuse Java library and can be used to translate any gene list into a meaningful graph. The specificity of the IBrowser plugin relies on a new "cell compartments-based layout" that makes use of a subset of the Gene Ontology to map gene products onto relevant cell compartments. This layout is particularly powerful for visual integration of heterogeneous biological information. Moreover, IBrowser is integrated into the TranscriptomeBrowser suite, which allows an easy communication with other tools, for instance to retrieve lists of genes that are frequently coexpressed in given conditions, thus creating context-specific views of the interactome and regulome.

IBrowser is intended both for biologists and bioinformaticians. On one hand, it is a graph-based knowledge browser, that is intended to provide new insight into any user-defined gene list. On the other hand it is also intended to fill the gap between heterogeneous genomic data and gene regulatory network analysis. In this regard, graphs produced inside IBrowser may be exported into Cytoscape and GINsim, a dynamic modeling software [32]. In the following sections we provide several examples underlying the benefits of this visualization tool for large gene set analysis.

## Implementation

We first used phylogenetic footprinting to predict regulatory elements in the human and mouse genomes. A dataset of 1,213 PWMs corresponding to mouse or human transcription factors was obtained from various sources (TRANSFAC 10.2, JASPAR 2010, UNIPROBE). The multiz28way (with hg18 as a reference) and the multiz30way (with mm9 as a reference) cross-species multiple alignments were obtained from UCSC [33]. We retained for analysis alignments flanking transcription start sites on both sides (-3000, 3000) of any RefSeq transcript and devoid of coding sequences. Sequences were scored following the commonly used formula [34]:

$$SCORE_{p,c}=\overset{W-1}{\underset{w=0}{\sum }}log\;2\left(\frac{P\left(seeing\;S_{p+w}\;at\;position\;w|PWM\right)}{P\left(seeing\;S_{p+w}\;at\;position\;w|Background\;model\right)}\right)$$

where *SCORE_p, c _*represents the PWM score for a PWM of length *W *in the DNA sequence of a species c between positions *p *and *p+W-1 *and *S_p+w _*represents the nucleotide observed at position *p+w*. The probability of observing each nucleotide under the background distribution was assumed to be 0.25. For each PWM *m*, a score threshold t_m _with p-value below 5.10^-5 ^was computed using matrix-distrib from RSAT ensuring high stringency of sequence scoring [35]. A sequence in the reference genome was considered as a putative TFBS if its score for PWM *m *at position *p *in the alignment was found above t_m _in human, mouse rat and dog. Each PWM was then linked to its corresponding transcription factors and putative targets. Information was stored in a MySQL relational database.

We also integrated several informations obtained from popular databases. Protein/DNA interactions (n = 174,168) derived from various genome wide analysis (e.g.; ChIP-on-chip, ChIP-seq and ChIP-PET) and encompassing interactions corresponding to 38 human TFs and 55 mouse TFs were obtained from the ChIP-X database. TFBS predictions were obtained from the present work (see below) and TFBSConserved UCSC track (n = 367,829 and n = 686,936 respectively). A set of regulatory interactions curated from the literature were obtained from LymphTF-DB (392 directed interactions) and OregAnno (1,991 interactions). Protein-protein interaction datasets were obtained from HPRD (n = 78,325), Reactome (n = 166,001) and IMEx (n = 110,578). Protein kinase-substrate relationships were retrieved from KEA (n = 14,084). Finally, miRNA/target relationships were obtained from TargetScan database predictions (n = 260,068). For all datasets, all identifiers were mapped onto Entrez Gene ids. This compendium of molecular interactions is available as flat files at: ftp://tagc.univ-mrs.fr/public/TranscriptomeBrowser/DB_Tables/.

InteractomeBrowser was developed using the Prefuse Java library which was modified according to our needs. InteractomeBrowser requires Java 1.6.

## Results and discussion

### TFBS predictions using comparative genomics

Although previous works have demonstrated the power of comparative genomics in defining novel regulatory motifs in human and mouse, few of them integrate the PWMs recently computed from protein binding microarray (PBM) experiments. Overall, restricting our analysis to promoter regions and using a set of 1,213 PWMs, we predicted TFBSs in 141,305 position-specific motifs of the mouse genome and 164,171 of the human genome. The median number of hits for any PWM was 117 in mouse (mean, 169; range, 3-2,317) and 122 in human (mean, 192; range, 6-2,678). The PWMs with highest number of hits correspond to Sp1 transcription factor (M00931, M00933, M00196) in both species (additional file 1, Figure S1). Sp1 binds GC-rich elements (consensus, GGGGCGGGGC) that are found in the promoter regions of a large number of genes [36]. As promoter regions are known to contain CpG islands we checked whether our approach could overestimate the number of targets for TF with high GC-content related PWMs. As shown in figure S1, this effect was essentially restricted to Sp1 and to a lesser extend to the Maz related PWM (consensus, RGGGAGGG). As expected, PWMs with high information content were most generally associated with fewer motifs (Figure S1, point size).

### Genes with highly conserved promoter regions mostly encode transcription factors

We next estimated the number of predicted regulators for each gene by computing the number of non-redundant PWMs associated with each gene. The number of PWMs that have a significant match in gene promoter regions range from 1 to 318 (median, 8; mean, 13.37) in mouse and 1 to 353 in human (median, 7; mean 13.17). Genes in the top 1% considering the number of regulators (eg; Lmo3, Foxp2, Bcl11a) were, as expected, invariably associated with highly conserved promoter regions. Moreover, functional annotation indicates that a very large proportion of these genes were transcription factors and genes related to development. Indeed, in mouse, enrichment analysis of the gene list (112 genes) using Fisher's exact test (with Benjamini and Hochberg correction) indicated a very strong enrichment for genes related to terms "Transcription factor" (PANTHER TERM; q-value, 1.3.10^-27 ^; 52 genes out 95 annotated), "pattern specification process" (GO biological process; q-value, 2.8.10^-13^; 19 genes out 78 annotated) or "neuron differentiation" (GO biological process; q-value,1.48.10^-09 ^; 18 genes out 78 annotated). Very concordant results were also observed for human (a summary of functional enrichment analysis using the ClueGO cytoscape plugin is provided in additional files 2 and 3, Figure S2 and S3) [37]. Actually, these results are in agreement with the work of Bejerano and collaborators that showed that ultraconserved elements of the human genome are most often found in genes involved in the regulation of transcription and development [38]. As a consequence our phylogenetic footprinting analysis predicts a higher number of motifs in the promoter regions of these genes. Although TFBS conservation in mammals has been previously analyzed in several papers, none of them, to our knowledge, reported this observation that may introduce a bias in the analysis. However, these ultraconserved regions may also be reminiscent of HOT (high-occupancy target) regions identified using ChIP-seq analysis in *Caenorhabditis elegans *and Drosophila [39,40]. Indeed, HOT regions have been shown to be significantly associated with "essential genes" (*i.e*.; having an RNAi phenotype of 100% larval arrest, embryonic lethality, or sterility) and genes related to growth, reproduction, and larval and embryonic development. However, we cannot rule out that these ultra-conserved regions may be also related to other mechanisms than regulation by site-specific TFs

### Biological relevance of the TFBS predictions

**Figure 1:**
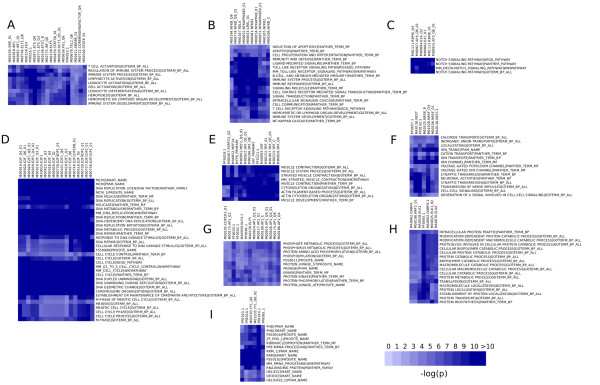
**Functional enrichment analysis of predicted targets**. Annotation terms obtained from various annotation databases were used to performed systematic annotation of all predicted target sets in the mouse. For each pair of term/PWM we computed Fisher's exact test p-value *f*. Each cell of a matrix with terms as row and PWM as column was filled with a score defined as *-log(f)*. (A-I) Representative biclusters found with BiMax are presented.

**Figure 2:**
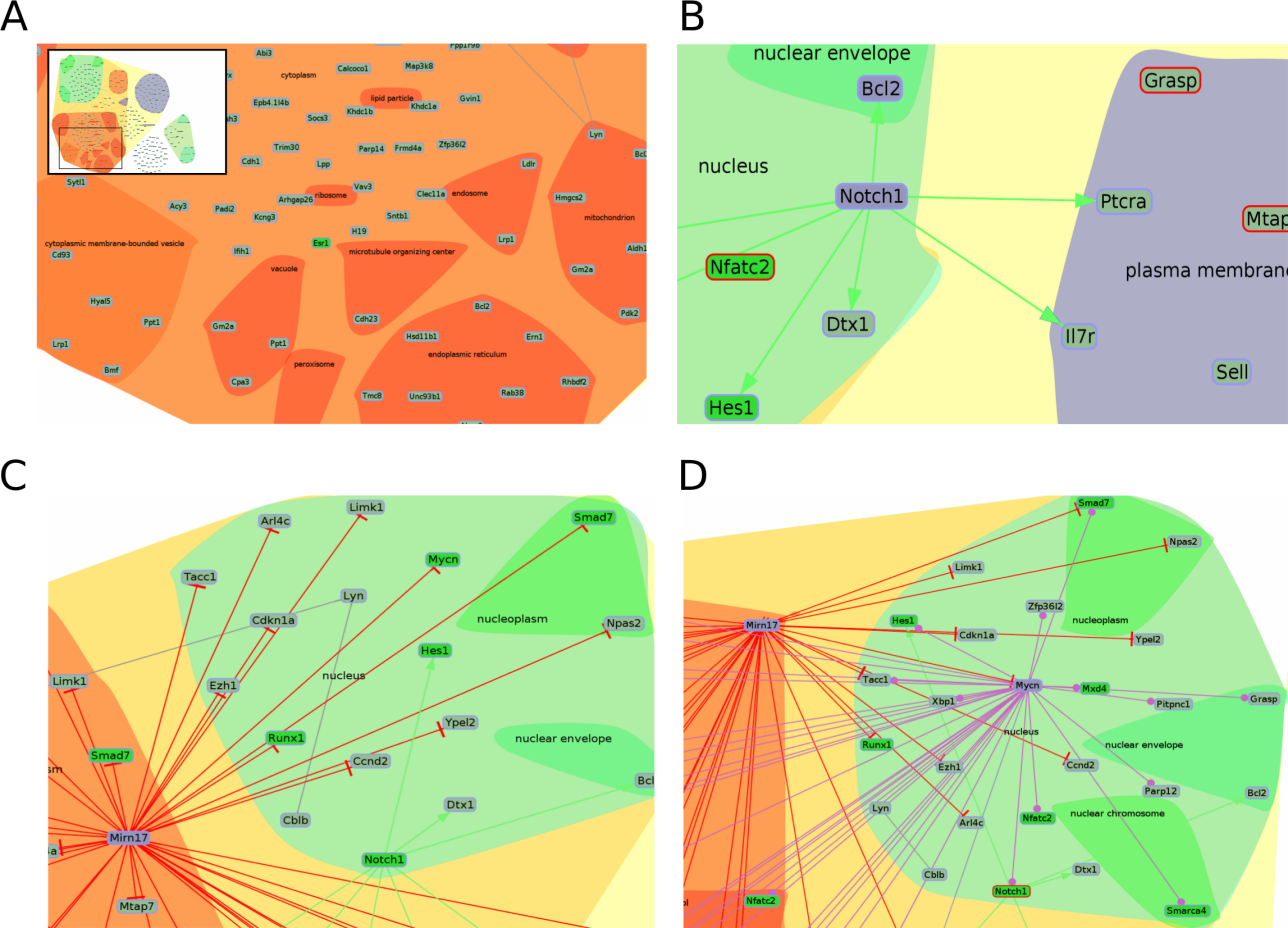
**The InteractomeBrowser plugin**. (A) A global and zoom-in view of InteractomeBrowser cell-compartment based layout. Zoom-in view shows some sub-cellular compartments together with node corresponding to gene products. Note that node corresponding to Esr1 appears as green, indicating that regulatory information is available for this gene. (B) Positive interactions (*i.e.; *activations) appear as green edges with normal arrowheads (here Notch1 is the source). (C) Negative interactions (*i.e; *repressions) appear as red edges with T-shaped arrowheads (here Mirn17 is the source). (D) Ambiguous interactions (whose repressive or activating status is unknown) appear as violet arrows with dot arrowheads (here with Mycn as source).

One criterion to assess the reliability of our predictions is based on the hypothesis that the overall functional properties of the predicted targets can be used to infer the biological processes in which TFs are involved. To test this hypothesis, we used annotation terms obtained from GO (biological process), KEGG, PANTHER, PFAM, SMART, PROSITE, and WIKIPATHWAYS databases and performed systematic annotation of all predicted target sets in the mouse [41]. For each pair of term/PWM we computed the Fisher's exact test p-value *f*. Each cell of a matrix with terms (n = 3,905) as row and PWM (n = 1,103) as column was filled with a score defined as *-log(f)*. We then searched for biclusters inside this matrix using "the binary inclusion maximal algorithm " (BiMax) [42]. Given the amount of information produced by this analysis, only some meaningful results will be presented and are summarized in Figure 1. Sites for PWM related to ETS (M00746, M00971, M00771, M00339, MA0136, M00658, M00678), STAT, IRF and RUNX (M00722) transcription factor families, known to contribute to pathogen responses, were significantly over-represented in genes annotated as "immune system process" and "lymphocyte activation" (Figure 1A). Sites for PWMs related to the Rel/NF-κB pathway were significantly associated with targets related to "induction of apoptosis", "Toll-like receptor signaling pathway" and, as expected to "NF-kappaB cascade" (Figure 1B). More subtle biclusters related to immune system were also found. As an example, RBPJK specific PWMs (M01112, M01111) were statistically significantly associated with terms "Notch signaling pathway". Although RBPJK is already known to be crucial in NOTCH signaling pathway, PWMs related to TCF3 (also known as E2A and E47) and AP-4 were also found in the same bicluster (Figure 1C). This observation is very consistent with the known role of these TFs in early B-cell differentiation, a development step for which Notch pathway is decisive [43,44]. As expected, a bicluster containing almost all E2F-related PWMs was also found. Finally, several biclusters related to "Muscle contraction", "Phosphorus metabolic processes", "Synaptic transmission", "Protein catabolic processes" and "Pre-mRNA processing" were also observed and are presented in Figure 2E-I. Altogether, these results highlight the biological relevance of the TFBS predictions and provides a systematic overview of putative regulatory interactions in human and mouse. These predictions have been termed "TBMC" (TranscriptomeBrowser Motif Conservation) and are available through the InteractomeBrowser plugin or as a bed file (See additional files 4 and 5).

### InteractomeBrowser: graph-based knowledge browser

The InteractomeBrowser application can be used to connect to our database in order to identify and analyze molecular interactions (See additional files 6 for a video tutorial). Available molecular interactions are derived from various sources: our predictions (TBMC) and numerous databases including ChIP-X, LymphTF-DB, OregAnno, HPRD, IMEx, Reactome, TargetScan and KEA. However, InteractomeBrowser may also accept additional interaction datasets that users can provide through a tabulated flat file.

InteractomeBrowser relies on a mixed graph that contains both directed and undirected edges, depicting various types of interactions ranging from proteins complex formation to transcriptional regulation. Thus nodes represent both genes and gene products.

InteractomeBrowser uses a subset of terms of the Cellular Component ontology (additional file, 7, figure S4) to map nodes onto a schematic and hierarchical view of cell compartments (users may choose to disable this option). As a consequence, each gene product may be represented by several instances (*e.g*.: one in the nucleus and one in the cytosol).

The nodes placement is controlled by a force-directed placement layout: the nodes are repulsive to each other, they are attracted to their respective compartments, and edges act like springs (the force-directed placement layout can be switched off or on at any moment through the "Display" menu). Once a graph has been drawn, one can easily add or delete nodes. InteractomeBrowser provides several filters that are intended to focus on the most interesting part of the network. Users can filter out orphan nodes and empty compartments. An option called "Hide intercompartmental edges" allows users to remove several unlikely edges of the network, notably those involving physical interactions between distant compartments (eg; an instance of gene A in the nucleus and an instance of gene B in the extracellular regions). When the mouse is over a node or an edge, corresponding information is provided in the "Infos" tab on the left side of the application. Right-clicking on a node opens a context menu, allowing users to (i) open the NCBI web page for this gene, (ii) add regulatory interactions involving this gene and other genes of the network, (iii) move the node to another compartment and (iv) connect to UCSC genome browser. The action menu provides other tools to expand the network: (i) add all the interactors of the selected genes or (ii) add common interactors of selected genes.

IBrowser can be used with any user-defined gene list, for examples genes of interest in a particular experiment. Additionally, the integration of this tool into the TranscriptomeBrowser suite facilitates the analysis of lists corresponding to pre-processed clusters of co-expressed genes stored in the database.

The next part of the result and discussion section demonstrates the use of InteractomeBrowser for retrieving molecular interactions in the context of thymocyte differentiation analysis.

### Case study: early T-cell development in mouse

The development of mature T cells from lymphoid progenitor cells involves a series of cell fate choices that direct differentiation. In the context of the Immunological Genome Project (ImmGen), M.W. Painter *et al *used rigorously standardized conditions to analyze expression levels of protein-coding gene in almost all defined T-cell populations of the mouse [45]. Using SAM analysis (FDR 15%), we selected a set of 281 genes repressed during the transition from thymic DN3 stage to DN4 stage. Careful analysis, indicated that this gene set was highly enriched in genes previously shown to be crucially involved during the first step of thymocyte development. This includes cell surface markers such as Il2ra/Cd25, and Il7r together with several transcriptional regulators, including Notch1, Smarca4/Brg1, Dtx1/Deltex1, and Hes1/Hry. More recently, Neilson *et al *identified specific miRNAs enriched at distinct stages of thymocyte development by deep sequencing [46]. The authors showed that transcripts of the mir17 family are up-regulated at DN4 stage and thus could be involved in the repression of DN3 specific messenger RNAs during DN3 to DN4 transition. We thus combined one member of the mir17 family, Mirn17/Mir17, with the mRNA gene list mentioned above. This gene list was provided as input to InteractomeBrowser. Figure 2A shows node placement according to cellular compartment. As shown in Figure 2A and 2B this layout is extremely useful to directly focus on genes of interest. Indeed, the nucleus subnetwork contains several regulators (*e.g; *Runx1, Notch1, Hes1 and Xbp1) some of them colored in green, indicating available regulatory interactions for the transcription factor in our database. Figure 2B shows that several genes (Dtx1, Hes1, Il7r and Bcl2) have been previously shown to be under the positive control of Notch1 (these curated informations are derived from LymphTF-DB). According to TargetScan predictions, Mirn17/Mir17 does not seem to target any component of the Notch pathway. In contrast, it is predicted to affect the expression of several transcription regulators including Mycn, Runx1, Smad7 and the H3K27 methyltransferase Ezh1 (by default miRNA are considered as having a negative effect on mRNA and thus edges appear as T-shaped arrows). Moreover, it may also control key components of the cell cycle machinery: Ccnd2 and Cdkn1a. Figure 2D shows informations available from ChIP-X database regarding Mycn. These informations are derived from a ChIP-seq experiment performed on mouse embryonic stem cells by Chen *et al *[47]. Note that according to these results, Mycn could target several transcription factors and thus play a key role during DN3 to DN4 transition. However, in this cellular context such results should be interpreted with caution since no large scale analysis of MYCN targets in DN3 Thymocytes has been reported so far. Among Mycn potential targets, Notch1, is one master switch of early to late thymocyte developmental transition. Thus, one could hypothesize that Mirn17/Mir17 may indirectly affect Notch1 by negatively regulating Mycn. Although, these hypotheses rely on predictions and on the assumption that Mycn binding to Notch promoter is effective in DN3 thymocyte, it clearly underlines the potential of this software in helping researchers to draw new hypotheses using data integration.

## Conclusions

InteractomeBrowser and its underlying approach can be compared to the Cerebral (Cell Region-Based Rendering And Layout) plugin of Cytoscape that also combines molecular interactions with a cell-compartment based layout [11].

But there are qualitative differences in the conception of Cerebral and InteractomeBrowser, which make the latest an interesting alternative for exploring networks.

On one hand, Cerebral uses a layered representation of the cell to create a "pathway-like" view of the network of interacting proteins. This layout thus provides a linear organisation of the network. On the other hand, the layout of InteractomeBrowser is based on a schematic view of the entire cell and displays the hierarchical structure of the underlying Gene Ontology subset as nested zones. First, this helps visually separating different parts of the network corresponding to different cellular localisations, as in Cerebral. But this is a more generic visualisation method, in the sense it does not restrict the visual message to an 'input-intermediates-output' mechanism such as in linear pathway diagrams. As a consequence it is suited for a more general study of various types of networks. Moreover, since visual zones correspond to Gene Ontology terms, this layout handles different levels of accuracy in the localisation of proteins: for instance a precisely-annotated protein might be placed in the zone corresponding to "endoplasmic reticulum", while a less well-annotated can be placed in the more generic, higher level zone "intracellular".

In Cerebral, each gene product is represented by one instance whose cell compartment may be defined by the user. In contrast, InteractomeBrowser displays, by default, several instances of a given gene product that may be placed in several cell-compartments according to informations provided by the GO Cellular-component ontology. Although this may lead to a more complex graph, it provides a more exhaustive presentation of current knowledge and may draw the attention of users to unexpected locations of gene products in the cells. The user may choose to delete some of these instances hence selecting *a posteriori *the most representative one.

The main benefit of InteractomeBrowser resides in its direct interaction with the database described in this report. Indeed, it provides a ready-to-use web-based service that requires only few manipulations to retrieve a network of interactions (see video tutorial provided as additional file). Notably, in addition to physical interactions it offers a unified access to miRNA targets and results from ChIP-Seq experiments derived from CHEA.

Presently, the data sources associated with the InteractomeBrowser plug-in are restricted to human and mouse. Indeed, one of the main objectives of InteractomeBrowser is to help users in creating regulatory maps to study human gene regulatory networks in physiological and pathological conditions. The choice of mouse as an additional organism supported by our database is a natural choice as it is a widely used model of human physiopathology. However, we are already planning to add new organisms in the near future.

As more and more experimentally validated interactions are available, we hope that this tool will prove very useful for researchers.

## Availability and requirements

InteractomeBrowser comes as a plugin for TranscriptomeBrowser and is available at: http://tagc.univ-mrs.fr/tbrowser/. Our database is updated on a regular basis. See additional files for a video tutorial.

• Project name: InteractomeBrowser

• Project home page: http://tagc.univ-mrs.fr/tbrowser/

• Operating system(s): Platform independent (Java)

• Programming language: Java

• Other requirements: Java > 1.6.X

• License: no license required

• Any restrictions to use by non-academics: none

## List of abbreviation used

PWM: Position Weight Matrices; GRN: gene regulatory network; GO: Gene Ontology; micro RNA: miRNA; TF: transcription factors; TFBS: transcription factor binding site; TBMC: TranscriptomeBrowser Motif Conservation.

## Competing interests

The authors declare that they have no competing interests.

## Authors' contributions

CL, AB, FL, CN, JI and DP conceived the project. CL, AB and FL developed the Java application. AB, CL and NBP developed the database. DP performed the TFBS analysis. DP, CN and JI supervised the project. DP wrote the manuscript. All authors read and approved the final manuscript.

## Supplementary Material

Additional file 1 **"Number of predicted motifs versus GC content of PWMs"**. Each point corresponds to the results obtained using one PWM on mouse genome. The name of a representative transcription factor for each PWM is displayed together with the PWM identifier (informations are separated using a pipe character). The size of the point is correlated with info content of the corresponding matrix). Click here for file

Additional file 2 **"Summary of functional enrichment analysis using ClueGO cytoscape plugin"**. We estimated the number of predicted regulators for each gene of the human genome by computing the number of non-redundant position-specific motifs associated with each genes. Genes in the top 1% regards to the number of regulators were used as input for the ClueGO plugin. Click here for file

Additional file 3 **"Summary of functional enrichment analysis using ClueGO cytoscape plugin"**. We estimated the number of predicted regulators for each gene of the mouse genome by computing the number of non- redundant position-specific motifs associated with each genes. Genes in the top 1% regards to the number of regulators were used as input for the ClueGO plugin. Click here for file

Additional file 4 **"TFBS predictions in the mouse genome"**. A bed file containing TFBS predictions in the mouse genome. 1 - chrom - The name of the chromosome. Fields contain the following informations: chromStart - The starting position of the feature in the chromosome; chromEnd - The ending position of the feature in the chromosome; name - PWM identifier and representative names; score - A score for the PWM hit; strand - Defines the strand - either '+' or '-'; gene id - The gene id of the target gene; geneSymbol- The genesymbol of the target gene. Click here for file

Additional file 5 **"TFBS predictions in the human genome"**. A bed file containing TFBS predictions in the human genome. 1 - chrom - The name of the chromosome. Fields contain the following informations: chromStart - The starting position of the feature in the chromosome; chromEnd - The ending position of the feature in the chromosome; name - PWM identifier and representative names; score - A score for the PWM hit; strand - Defines the strand - either '+' or '-'; gene id - The gene id of the target gene; geneSymbol- The genesymbol of the target gene. Click here for file

Additional file 6 **"InteractomeBrowser functionalities"**. Contains a web link to a screencast showing basic use of InteractomeBrowser plugin. Click here for file

Additional file 7 **"Subset of Gene Ontology used for the cell compartment-based layout"**. Hierarchical structure of the subset of Gene Ontology used in InteractomeBrowser for the cell compartment-based layout. Colors highlight the main compartments. Click here for file
