# Supplementary figures and images for: TranscriptomeBrowser 3.0: introducing a new compendium of molecular interactions and a new visualization tool for the study of gene regulatory networks
Source: BMC Bioinformatics. 2012 Jan 31;13:19. doi: 10.1186/1471-2105-13-19 (PMC3395838; doi:10.1186/1471-2105-13-19)

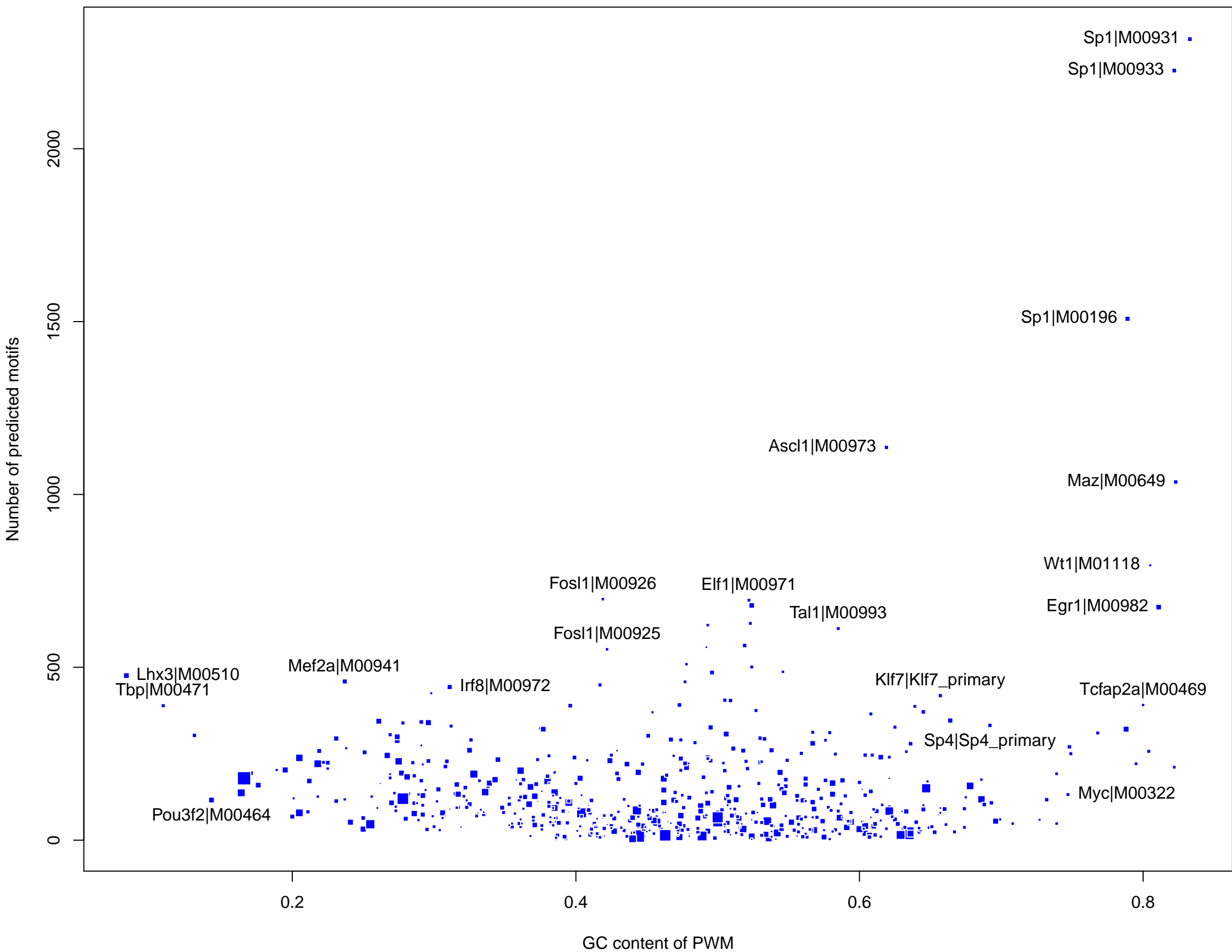

Supplement: Additional file 1 — "Number of predicted motifs versus GC content of PWMs". Each point corresponds to the results obtained using one PWM on mouse genome. The name of a representative transcription factor for each PWM is displayed together with the PWM identifier (informations are separated using a pipe character). The size of the point is correlated with info content of the corresponding matrix). [file 1471-2105-13-19-S1.PDF]

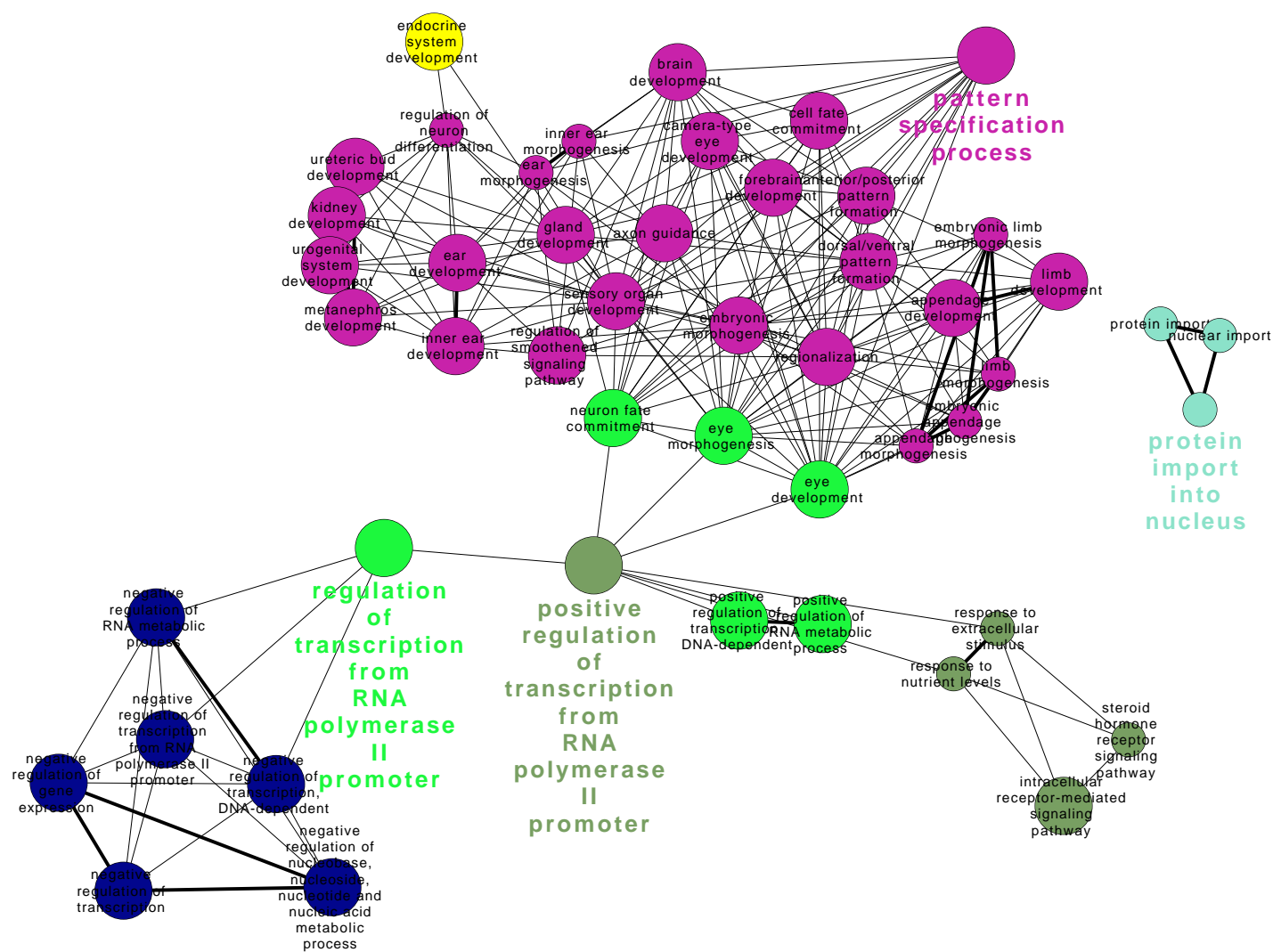

Supplement: Additional file 2 — "Summary of functional enrichment analysis using ClueGO cytoscape plugin". We estimated the number of predicted regulators for each gene of the human genome by computing the number of non-redundant position-specific motifs associated with each genes. Genes in the top 1% regards to the number of regulators were used as input for the ClueGO plugin. [file 1471-2105-13-19-S2.PDF]

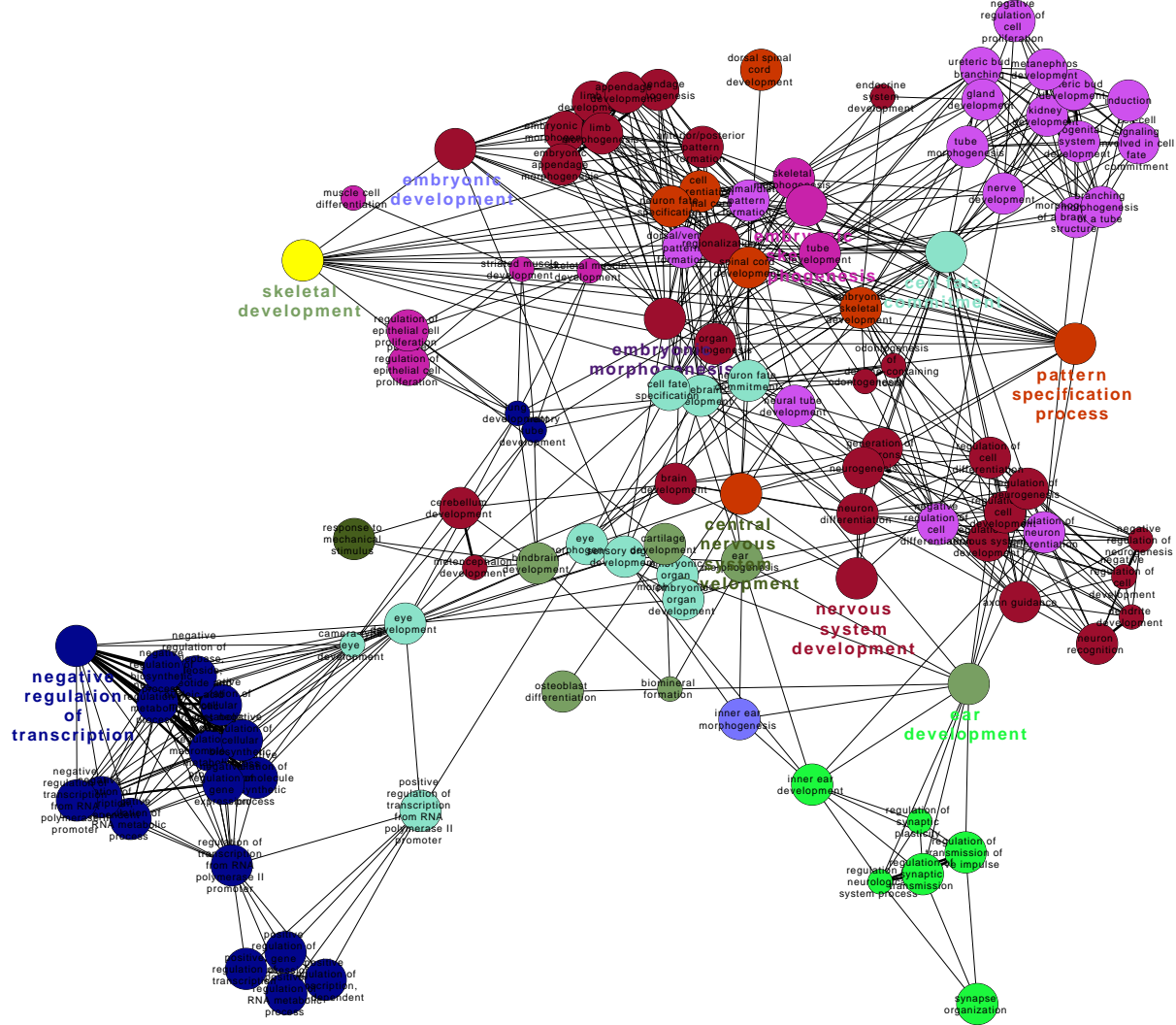

Supplement: Additional file 3 — "Summary of functional enrichment analysis using ClueGO cytoscape plugin". We estimated the number of predicted regulators for each gene of the mouse genome by computing the number of non- redundant position-specific motifs associated with each genes. Genes in the top 1% regards to the number of regulators were used as input for the ClueGO plugin. [file 1471-2105-13-19-S3.PDF]

# cellular component

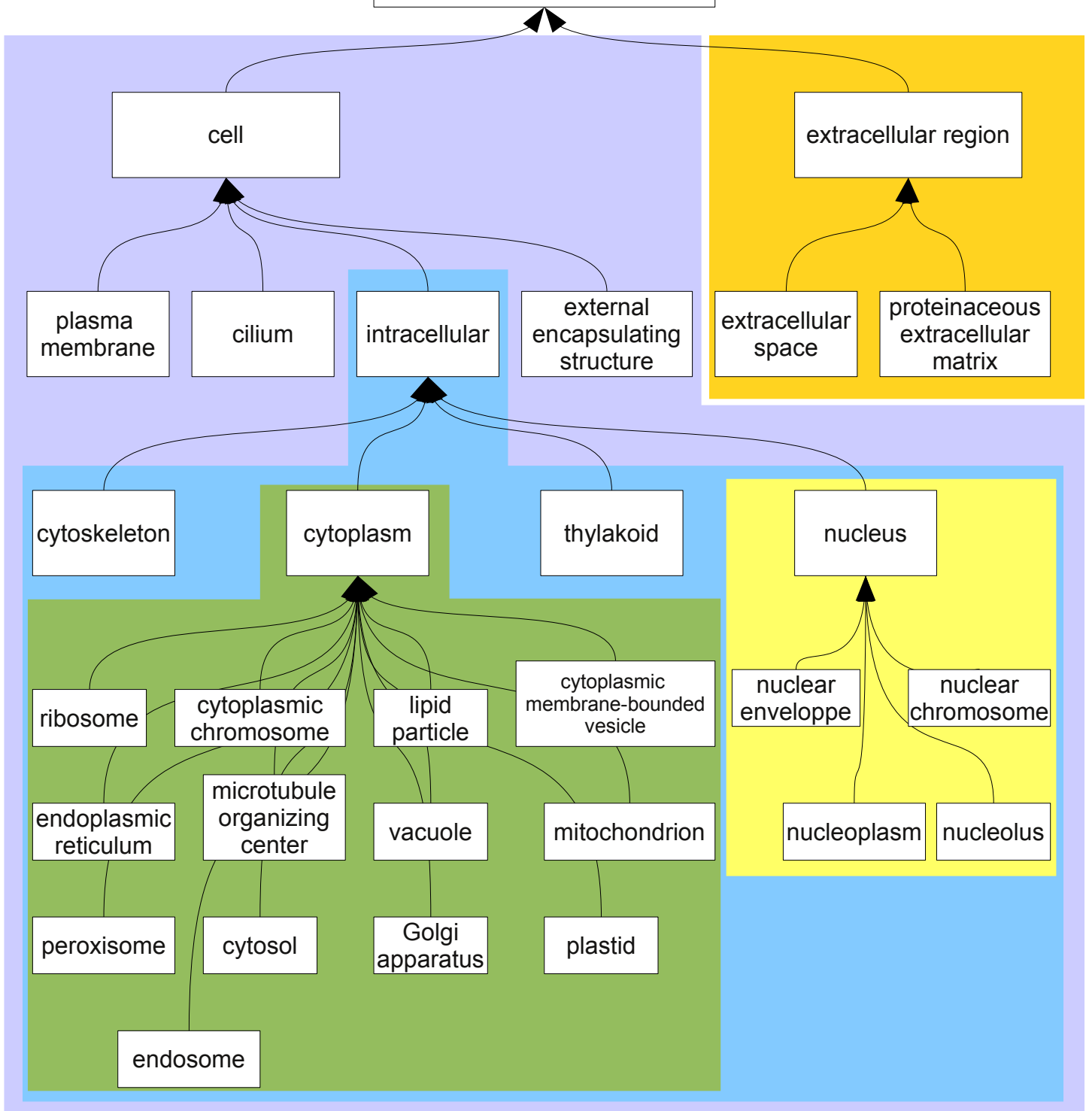

Supplement: Additional file 4 — "TFBS predictions in the mouse genome". A bed file containing TFBS predictions in the mouse genome. 1 - chrom - The name of the chromosome. Fields contain the following informations: chromStart - The starting position of the feature in the chromosome; chromEnd - The ending position of the feature in the chromosome; name - PWM identifier and representative names; score - A score for the PWM hit; strand - Defines the strand - either '+' or '-'; gene id - The gene id of the target gene; geneSymbol- The genesymbol of the target gene. [file 1471-2105-13-19-S4.PDF]
