# Supplementary material for: TranscriptomeBrowser 3.0: introducing a new compendium of molecular interactions and a new visualization tool for the study of gene regulatory networks
Source: BMC Bioinformatics. 2012 Jan 31;13:19. doi: 10.1186/1471-2105-13-19 (PMC3395838; doi:10.1186/1471-2105-13-19)
Supplement: Additional file 7 — "Subset of Gene Ontology used for the cell compartment-based layout". Hierarchical structure of the subset of Gene Ontology used in InteractomeBrowser for the cell compartment-based layout. Colors highlight the main compartments. [file 1471-2105-13-19-S7.DOC]

A video describing basic functionalities of InteractomeBrowser is available at:

<http://www.youtube.com/watch?v=SxOBmCP1G1A>
